# The Importance of Post-Mortem Investigations in Stillbirths: Case Studies and a Review of the Literature

**DOI:** 10.3390/ijerph19148817

**Published:** 2022-07-20

**Authors:** Carmen Scalise, Fabrizio Cordasco, Matteo Antonio Sacco, Pietrantonio Ricci, Isabella Aquila

**Affiliations:** Institute of Legal Medicine, Department of Medical and Surgical Sciences, University “Magna Graecia” of Catanzaro, 88100 Catanzaro, Italy; scalisecarmen@libero.it (C.S.); cordasco@unicz.it (F.C.); matteoantoniosacco@gmail.com (M.A.S.); ricci@unicz.it (P.R.)

**Keywords:** forensic science, stillbirth, placenta histology, forensic autopsy, intrauterine fetal death, placental examination

## Abstract

Stillbirth has an important economic and social impact, though it remains “inexplicable” in many cases. We report the analysis of 11 cases of intrauterine fetal death carried out through a retrospective study conducted in the period between 2014 and 2017. The purpose of the study is to quantify the contribution of the autopsy and placental examination in identifying the cause of stillbirths. For each case, the medical record was analyzed with the relative maternal and partner data, the results of the external fetal and autopsy examination as well as the macroscopic and histological placental examination. The peak of stillbirth was found in a maternal age group between 30 and 39 years, below the 32nd week and above the 37th week of gestation. The results obtained from the clinical history and external fetal examination make it possible to trace the cause of death in only 18.2% of cases. By adding to these data, the results of the fetal autopsy and the placental examination, it is possible to establish the cause of death in 90.9% of cases. The most frequent abnormalities found in the placenta and cord were short or hypercoiled cord, umbilical cord vascular thrombosis, turns around the neck or stretching of the funiculus, placental infarction and placental insufficiency; also, amniotic fluid abnormalities, such as suspected oligohydramnios and chorioamniositi, were found. The accurate analysis of post-mortem placental and fetal examination is essential to reduce the number of unresponsive intrauterine fetal deaths. Determining the cause of fetal death must help clinicians and parents in better management and care in future pregnancies.

## 1. Introduction

Stillbirth represents a social and economic problem all over the world. Stillbirth occurs at 6 in 1000 pregnancies in the United States and more than 3000 babies are stillborn in the United Kingdom every year [1,2]. One-third of fetal deaths occur after 37 weeks of gestation in apparently healthy infants. This unexpected event generates discomfort and frustration in parents and doctors, and stillbirths remain unanswered in 25–60% [3].

This document aims to focus on the importance of fetal autopsy and placental examination in the study of the causes of intrauterine fetal deaths. In fact, maternal and gestational medical history alone in most cases is not sufficient to determine the cause of death and still today, many cases are unanswered. The pathologist plays an essential role in the management of intrauterine fetal deaths. Post-mortem fetal and placental examination should be common practice in the study of fetal deaths.

This work will impact the scientific community by demonstrating that the purpose of post-mortem investigation is to determine the causes of death and its mechanisms, as well as to help both healthcare professionals and parents to understand the complex dynamics of intrauterine fetal death by reducing the number of unexplained cases and, thus, manage future pregnancies. For this reason, we want to highlight how the results obtained by the pathologist must always be integrated with clinical information in order to have a complete picture so as to identify the cause of the unfortunate event.

The WHO defines stillbirth as the loss of the fetus after the 22nd week of gestation or, in the case of unknown dating, for a birth weight greater than or equal to 500 g. The economic, social, health and family impact of intrauterine fetal deaths in the world is devastating. The intrauterine fetal death rate ranges from 2.1 per 1000 to 40 per 1000 worldwide, with a total of 2.6 million cases each year. In developed countries, about 1 in 200 pregnancies result in stillbirths [4]. Poor social groups are the most affected; 98% of stillbirths occur in low- and medium-developed countries and more than two-thirds occur in rural environments. About 1.2 million stillbirths occur during labor and delivery, and in most cases, these are full-term babies who could be saved with appropriate quality care [5]. In Italy, intrauterine fetal deaths were 30.96 per 1000 in 1950 and they have steadily decreased 10-times over 50 years. The progressive decrease from year to year in the average death rate in utero worldwide, on the other hand, is slower (about 1.1% between 1995 and 2009) than that of maternal and neonatal mortality (2.3%) [6]. A recent Japanese study showed that the causes of stillbirths have changed over a few years [7]. The study, considering 2001–2007 and 2008–2014 as observation periods, underlines that in the second period of time, the causes of stillbirths are more attributable to placental and umbilical cord pathologies (which have increased), compared to fetal anomalies, which, instead, have been reduced. These changes could be attributed to better management of pregnancy and the practice of therapeutic abortions following prenatal screening, which allowed for early diagnosis of fetal pathologies. On the other hand, there has also been an increase in pregnancies that get through medically assisted procreation techniques, which, according to various data, tends to increase the risk of placenta previa (RR 3.71), placental abruption (RR 1.83), polyhydramnios (RR 1.74) and oligohydramnios (RR 2.14) [8,9]. Placental and umbilical cord pathologies are frequent conditions, but little linked to the management of pregnancy, often asymptomatic and, therefore, difficult to diagnose early and nowadays, it is complex to implement prevention protocols. To understand the difficulty in the nosological classification of the issue, more than thirty classification systems have been introduced since 1954, with completely dissimilar purposes, approaches, definitions, levels of complexity and reference guidelines. In many cases, the death of the fetus is not followed by an accurate genetic, placental and autopsy investigation, but only by the closure and frustration of the parents, who have no answer or useful information for upcoming pregnancies. In addition, stillbirth has always represented a challenge for the physician who often deals with a situation that rapidly changes in a pregnancy with physiological course and with a newborn without obvious malformations. The clinical history and the investigations carried out during pregnancy hardly ever lead to a diagnosis of certain death.

## 2. Materials and Methods

This is a retrospective study on all cases of stillbirth with childbirth at 23rd week at the “Magna Graecia” University of Catanzaro from 2014 to 2017, for a total of 11 cases.

The sample analyzed is represented by cases of intrauterine fetal death, i.e., all “stillbirths” at the time of expulsion occurring beyond the 22nd week of gestation. For each case, the maternal clinical history with related clinical events that occurred during pregnancy was evaluated; clinical data relating to the partner; the external fetal examination (and possibly also the autopsy examination) and both macroscopic and microscopic placental examination, through histological examination. Cases that did not have these elements were not included in the study.

### 2.1. Clinical Investigations

Clinical history of the pregnant woman and her partner was investigated by considering the presence of previous or ongoing infections during pregnancy, abnormalities of the karyotype or hereditary genetic diseases, risk factors for stillbirth of the mother such as gestational diabetes, obesity, arterial hypertension, eclampsia, cigarette smoke. Infectious factors were examined in the mother’s medical history through evaluation of laboratory investigations performed during pregnancy. In particular, infectious diseases that cause TORCH syndrome, were investigated, i.e., Toxoplasma gondii, rubella, Citomegalovirus and Herpes simplex [10,11,12].

### 2.2. Pathological Investigations

All cases underwent a fetal autopsy examination.

Fetal autopsies were performed through a standard protocol including anthropometric profile, internal autopsy examination, macroscopic and microscopic examination of the organs and placenta.

The external examination must include the phenotypic assessments, the state of conservation of the skin, the assessment of any injury, the evaluation of hypostasis, the mobility of the bones of the cranial vault and joints of the limbs, and the assessment of any deformities.

Autopsy allows the macroscopic evaluation of the organs and any anomalies. It must be associated with the withdrawal of biological fluids for toxicological (blood, urine, bile), virological or microbiological (blood, cerebrospinal fluid) purposes. A sample was taken from each organ for histopathological examination.

The causes of death were divided into maternal, fetal, placental or traumatic, and were analyzed according to the classification of stillbirth by relevant condition at death (ReCoDe) [13].

### 2.3. Placental Investigations

All cases underwent systematic gross and histological placental examination. This examination included recording of placental weight, membrane insertion, umbilical cord length measurement and wrapping pattern. An analysis was carried out for any abnormalities of the placenta (placental infarction, placental insufficiency) and its annexes as anomalies of the umbilical cord (true gyrus, thrombosis of the vessels), and abnormalities of the amniotic fluid (oligodramnios polyhydramnios). The entire placenta after delivery was fixed in fixation fluid (10% buffered formalin).

Placental abnormalities, instead, were evaluated according to the Stockholm classification (direct cause, major contribution, minor contribution and not involved in determining intrauterine fetal death).

## 3. Results

**Figure 1:**
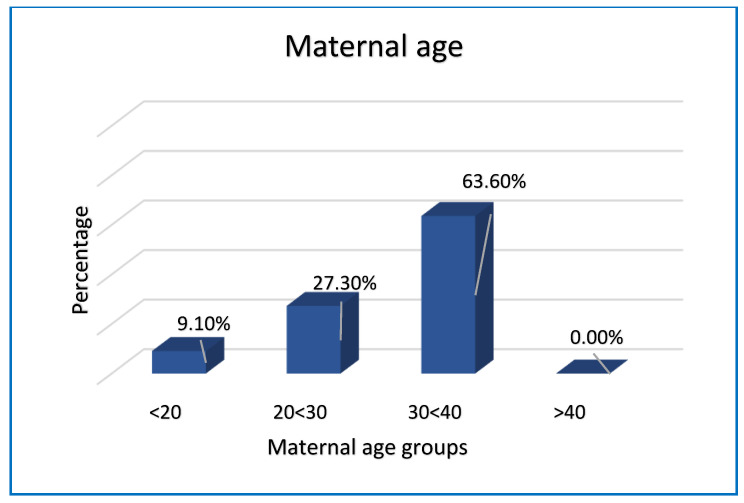
Maternal age and stillbirth.

The maternal clinical history allowed the division of maternal age into bands. In 9.1% of cases, the mother was under the age of 20; 27.3% of cases fell within the age group between 20 and 29 years; between 30 and 39 years, 63.6% of cases was included and, in the analyzed data, no mother was older than or equal to 40 years (Figure 1). The average age was 30.4 years.

In 81.8% of cases, these were first-time pregnancies, multiple in 18.2% of cases.

**Figure 2:**
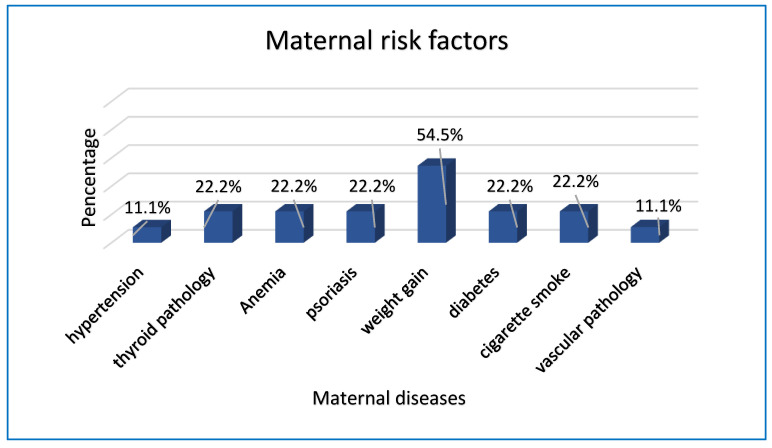
Maternal risk factors.

From the clinical history, we found that out of 11 cases, in 9, there were two or more risk factors for pregnancy. In 11.1% of cases, there were arterial pathologies and circulatory failure; in 22.2% of cases, thyroid diseases, microcytic anemia, psoriasis, gestational diabetes or cigarette smoking during pregnancy; in 33.3% of cases, there was a significant weight increase, beyond the normal values indicated by the guidelines (>7 kg but <9 kg). These pathologies were all adequately treated pharmacologically according to the guidelines (Figure 2). No cases of infectious diseases that cause TORCH syndrome were found.

Another important aspect of data was anamnesis: in three cases, the pregnancy was complicated by threats of abortion, adequately treated with the Prontogest. However, in two of these cases, no maternal risk factors were identified.

Furthermore, from the medical records, out of 11 cases, 3 carried out induced birth, in 6 cases, the delivery took place via emergency caesarean section and, finally, in 2 cases, a caesarean section was used after having performed a previous induction.

We also observed that the eight cases of stillbirth, which occurred from the 37th week of gestation onwards, had a normal cardiotocographic pattern and that the alterations appeared suddenly, forcing the completion of the childbirth.

**Figure 3:**
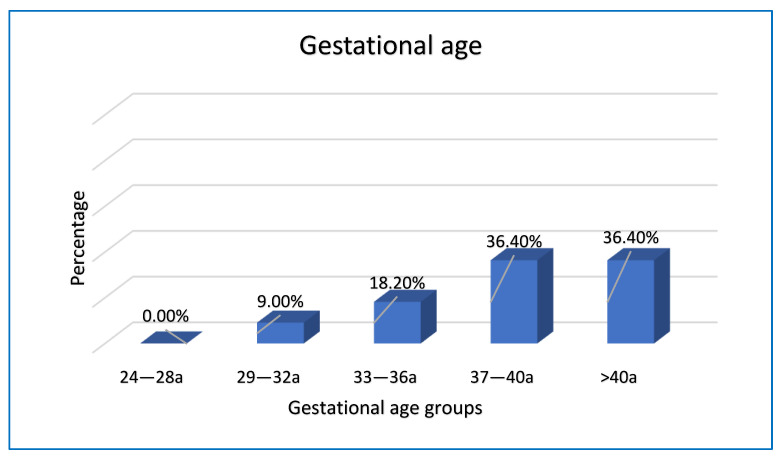
Gestational age and stillbirth.

We did not find cases of stillbirth in the gestational age group between the 24th and 28th week; 9% of cases were between the 29th and 32nd week of gestation; 18.2% of cases in the gestational age group between the 33rd and 36th week; 36.4% of cases were found both in the range between 37th and 40th week, and in the range that includes pregnancies over the 40th week of gestation (Figure 3).

**Figure 4:**
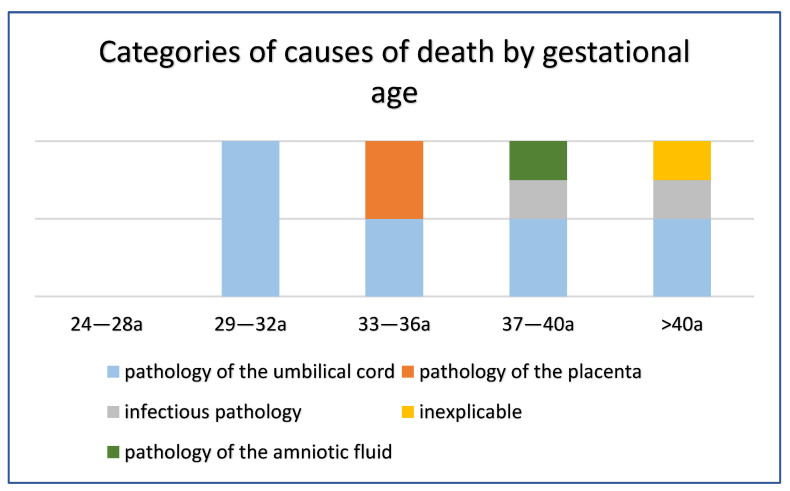
Categories for cause of death by gestational age.

Considering the cause of intrauterine fetal death in relation to the gestational age group, umbilical cord disorders (including hypercoiling, umbilical cord vascular thrombosis, turns around the neck) represented the cause of death in all cases falling within the range between 29th and 32nd week; in the range between the 33rd and the 36th week of gestation, in 50% of cases, the cause involved the umbilical cord; in the remaining 50% of cases, the placenta. Always in 50% of cases, the cause was referred to the pathology of the umbilical cord in the gestational age group between the 37th and 40th week, together with 25% of cases where the cause was of infectious origin and 25% of cases, the cause of which is the amniotic fluid; finally, above the 40th week, 50% of cases were attributable to the pathology of the umbilical cord, 25% of infectious origin and 25% defined as unexplained stillbirth (Figure 4).

By using the ReCoDe classification [13], it was possible to deduce that, among the 11 cases, there were no causes attributable to fetal alterations, intrapartum complications, trauma, uterine morphological abnormalities or maternal causes.

On the other hand, seven cases fell within the pathology of the umbilical cord, such as short and hypercoiled cord, thrombosis of the vessels, turns around the neck or stretching of the funiculus; two cases fall under amniotic fluid abnormalities, such as suspected oligohydramnios and chorioamniositis; one case in placental pathology due to metabolic disorder, including a harmful event of metabolic origin (such as gestational diabetes) and one case in the category of unexplained stillbirth (Table 1).

The Stockholm classification, instead, allows one to evaluate whether placental abnormalities are a direct cause, have acted more or less or even if they are not the cause of stillbirth.

In this study (Table 2), only one case showed placental pathology as a direct cause of stillbirth; seven cases where it made a major contribution; three cases where it had a minor contribution; in one case, placental changes were not considered to be causes of stillbirth.

The cause of stillbirth, for each case of the study, was obtained through three phases, adding to each phase, an element for its determination and evaluating the corresponding percentage of stillbirth sine causa.

A careful analysis of clinical events and external fetal examination allows one to identify the cause of intrauterine fetal death in only 18.2% of cases. The percentage of unexplained stillbirths would, thus, be 81.8%. However, adding the results obtained from macroscopic and microscopic examination of the placenta to these data, the cases with explainable death increase from 18.2% to 81.8%.

Adding to the previous ones, the results obtained from the fetal autopsy do not add important information to determine the cause of death, even if it is useful to confirm the diagnosis. In fact, the fetuses analysed on external examination were all of normal shape (four males and seven females), with body weight, head circumference and length consistent with gestational age. In only one case, there was an intrauterine growth retardation (IUGR), probably caused by the suspected oligohydramnios. For another case, however, there is no information regarding the internal examination of the fetus and organ, as the autopsy examination was not authorized. Unexplained deaths, despite the implementation of a standard protocol, which provides for the inclusion of all the information obtained from the three phases, remain 9.1%.

## 4. Discussion

The results of our study show that the maternal age group between 30 and 39 years is the one where most cases are concentrated (63.6%). Primiparous pregnancies and the male sex of the fetus are most affected by stillbirth, with 81.8% and 63.6%, respectively.

**Figure 5:**
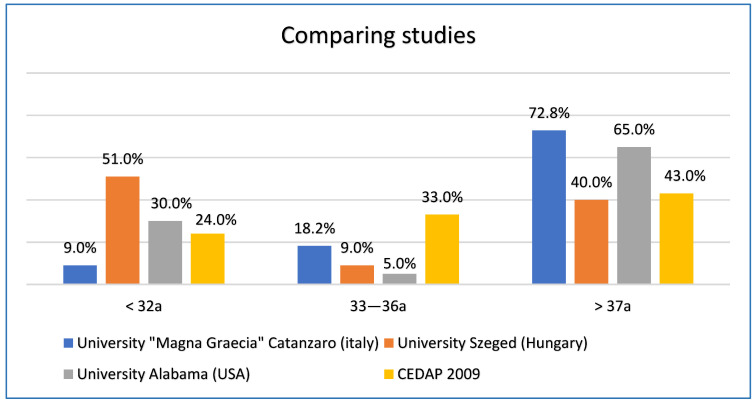
Comparing studies.

Intrauterine fetal deaths have a peak starting from the 37th week of gestation (72.8% of cases) and these data are in agreement with the national and international literature (Figure 5). Indeed, a retrospective study, published in 2014 [14] and conducted on 140 cases of stillbirth analyzed at the University of Szeged in Hungary, in the period between 1996 and 2010, shows how stillbirths have a peak in the 31st week, which then tends to decrease until the 37th week, and then increase again near the end of the pregnancy.

Another study, carried out at the University of Alabama in Birmingham [15], published in 2017, where cases of intrauterine fetal death from 2009 to 2013 were analyzed in Native American, non-Hispanic white, non-Hispanic black and Hispanic women, demonstrated how the risk of fetal death changes according to gestational age and maternal ethnicity. Indeed, in black non-Hispanic and white non-Hispanic women, the greatest risk occurs before the 36th week. On the contrary, for Native American women, the stillbirth peak occurs in week 32, then decreases from week 33 and increases again from week 37. Furthermore, the results of the CEDAP 2009 (Certificate of Childbirth Assistance) [16] published by the Ministry of Health, show that the highest percentage of cases of stillbirth in Italy is also after the 37th week.

A review, conducted on 41 studies in the literature [17], both European and American, where placental changes and their related appendages are evaluated as a cause of intrauterine fetal death, published in 2014 and implemented at the Research Center for Maternal and Fetal Health of the University of Manchester, established that chorioamniositis (10.6% of cases), anomalies of the umbilical cord (81% of cases) and thrombosis of the vessels of the funiculus (8.4% of cases) represent the major causes of stillbirth. This figure is in agreement with our results. Indeed, out of 11 cases of stillbirth, in 9 cases, one or more of these pathological pictures coexist and cause death: chorioamniositis in 9.1% of cases, anomalies of the umbilical cord in 63.6% of cases, thrombosis of the vessels in 18.2% of cases.

**Figure 6:**
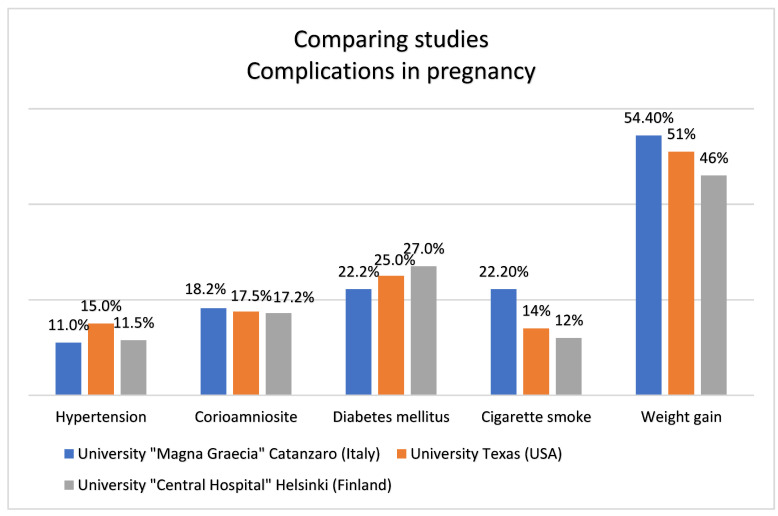
Comparing studies.

Weight increasing in pregnancy, beyond the normal permitted values, is the maternal risk factor most found in the anamnestic examination in over half of the cases (54.5% of cases). Furthermore, almost 90% of pregnant women reported one or more risk factors in addition to weight gain (gestational diabetes, hypertensive peaks, circulatory insufficiency) in their medical history and these data are in agreement with a review of the evidence in the literature, published in 2007, thanks to the collaboration of many American universities [18]. Indeed, weight increasing heightens the risk of developing gestational diabetes by 6.3% compared to the normal population, increases the risk of developing hypertension by five-times and also increases the likelihood of emergency caesarean section. The same review estimated that hypertension complicates between 10 and 20% of pregnancies and the infections complicate between 10 and 25% of pregnancies. According to these considerations, in our study, hypertension is present in 11% of cases and chorioamniositis in 18.2% of cases. The results in our study also agree with a study published in 2010 by the “Central Hospital” University of Helsinki, Finland [19]. In this study, hypertension complicates pregnancy between 8 and 15%; chorioamniositis between 15 and 20% (Figure 6).

The ReCoDe classification was essential to reduce the number of unexplained fetal deaths, by demonstrating that in 90.9% of the cases analyzed, the cause of stillbirth was due to placental pathologies or its appendages.

The Stockholm classification, on the other hand, allowed us to evaluate the contribution of placental pathology as a cause of fetal death: in 72.7% of cases, placental abnormalities made a direct or greater contribution to causing intrauterine fetal death. These data are fundamental to understand how, even if the placenta is not primarily involved in determining fetal death, it is subsequently involved when the pathology affects the placental adnexa. Indeed, many pathologies of the umbilical cord or/and of the amniotic fluid cause, secondarily, placental insufficiency, hence, fetal suffering and finally stillbirth. Our study also shows how, thanks to the placental examination, it was possible to reduce stillbirth sine causa from 81.8% to 9.1. It should also be emphasized that the external fetal examination, even if not associated with other investigations, can lead to an incorrect diagnosis of death.

## 5. Conclusions

Significant conclusions can be drawn from all the information carried out from the study and compared from the literature. Macroscopic and microscopic placental examination is essential to determine the cause of fetal death. It is not ethically and scientifically correct to report a diagnosis of death without having integrated all the results from the various investigations, from the clinic to the autopsy examination. The study, according to the international literature, indeed, has shown that the lack of data regarding the placenta and its appendages often leads to an erroneous diagnosis of death. It is also essential to always implement a careful maternal clinical history evaluation, since risk factors, although not often direct causes of stillbirth, are constantly present in these cases. This allows us to conclude that, in some way, they play a non-marginal role in creating metabolic imbalances, which subsequently affect placental activity. Although all the investigations to verify the cause of stillbirth were carried out as per standard protocol, in a variable percentage of cases, this is defined as “inexplicable”. This concept highlights how, even today, medical knowledge on the matter is not yet sufficient to be able to have a certain diagnosis for each case of fetal death.

Finally, all these considerations have the aim not only to explain the events that occurred in full truth to parents, but also to offer them adequate prevention and surveillance for subsequent pregnancies.

## Figures and Tables

**Table 1:** ReCoDe Classification.

*ReCoDe Classification*		
Causes of Stillbirth	Number of Cases	Percentage
***Fetal***	0	0.00%
***Umbilical cord***	7	63.6%
***Placental***	1	9.10%
***Amniotic fluid***	2	18.20%
***Uterus***	0	0.00%
***Maternal***	0	0.00%
***Intrapartum***	0	0.00%
***Trauma***	0	0.00%
***Inexplicable***	1	9.10%

**Table 2:** Stockholm Classification.

*Stockholm Classification*		
Category	Cases	Percentage
***Direct death injury***	1	9.10%
***Injury contributing greater than death***	7	63.60%
***Minor injury contributing to death***	2	18.20%
***Injury not causing death***	1	9.10%

## Data Availability

Not applicable to this article, as no datasets were generated.
